# Dietary plasmalogen increases erythrocyte membrane plasmalogen in rats

**DOI:** 10.1186/1476-511X-11-161

**Published:** 2012-11-21

**Authors:** Shiro Mawatari, Toshihiko Katafuchi, Kiyotaka Miake, Takehiko Fujino

**Affiliations:** 1 Institute of Rheological Function of Food, 2241 Kubara, Hisayama-chou, Kasuya-gun, Fukuoka, 811-2501, Japan; 2 Department of Integrative Physiology, Graduate School of Medical Science Kyushu University, Fukuoka, 812-8582, Japan; 3 Central Research Institute, Marudai Food Co. Ltd, Osaka, Japan; 4 Institute of Rheological Function of Food, 2241 Kubara, Hisayama-chou, Kasuya-gun, Fukuoka, 811-2501, Japan

**Keywords:** Dietary plasmalogen, Zucker diabetic fatty rat, Wistar rat, Erythrocyte phospholipids, Plasma phospholipids

## Abstract

**Background:**

Many disorders with plasmalogen deficiency have been reported. Replenishment or replacement of tissue plasmalogens of these disorders would be beneficial to the patients with these disorders, but effects of dietary plasmalogen on mammals have not been reported.

**Methods:**

Plasmalogens were purified from chicken skin. The purified plasmalogens consisted of 96.4% ethanolamine plasmalogen (PlsEtn), 2.4% choline plasmalogen (PlsCho) and 0.5% sphingomyelin (SM). A diet containing 0.1% the purified plasmalogens (PlsEtn diet) was given to rats. Relative composition of phospholipids was measured by a high performance liquid chromatography (HPLC) method that can separate intact plasmalogens and all other phospholipid classes by a single chromatographic run.

**Results:**

The PlsEtn diet given to Zucker diabetic fatty (ZDF) rats for 4 weeks caused decreases of plasma cholesterol and plasma phospholipid as compared to control diet. The other routine laboratory tests of plasma including triacylglycerol, glucose, liver and renal functions, albumin, and body weight were not different. Relative compositions of erythrocyte PlsEtn and phosphatidylethanolamine (PE) increased, and that of phosphatidylcholine (PC) decreased in PlsEtn diet group. The PlsEtn diet given to normal rats for 9 weeks again caused decrease of plasma cholesterol and phospholipid, and it induced increase of relative composition of PlsEtn of the erythrocyte membrane. The other routine laboratory tests of plasma and body weight were not different.

**Conclusions:**

Dietary PlsEtn increases relative composition of PlsEtn of erythrocyte membranes in normal and ZDF rats, and it causes decreases of plasma cholesterol and plasma phospholipids. Dietary PlsEtn for 9 weeks seemingly causes no adverse effect to health of normal rats.

## Background

Plasmalogens are glycereophospholipids characterized by the presence of vinyl ether bond at the sn-1 position of glycerol backbone and an ester bond at the sn-2 position. The sn-1 position is most commonly linked to C16, C18, or C18:1 fatty alcohols and the sn-2 position typically occupied by an polyunsaturated fatty acid, specifically arachidonic acid (ARA) or docosahexaenoic acid (DHA) [1-5]. Plasmalogens are found in almost all mammalian tissues and constitute about 18% of the total phospholipids in cell membranes, with ethanolamine plasmalogen (PlsEtn) much more abundant than choline plasmalogen (PlsCho) except heart and skeletal muscle [1-5]. Plasmalogens are not only structural component of mammalian cell membrane and a reservoir for second messengers, but also may involve membrane fusion, ion transport and cholesterol efflux [1-5]. The vinyl ether bond at the sn-1 position makes plasmalogens more susceptible to oxidative stress than the corresponding ester bonded glycerophospholipids, plasmalogens may also act as antioxidants, protecting cells from oxidative stress [1-5].

Plasmalogen biosynthesis starts in peroxisome, and the inherited human peroxisomal disorder, rhizomelic chondrodysplasia punctata (RCDP), shows deficiency of tissue plasmalogens in multiple organs and causes severe disorders of multiple organs such as bone, brain, lens, kidney and heart [2-5]. These disorders of RCDP may be a direct consequence of plasmalogen deficiency and indicate importance of plasmalogens in human body. On the other hand, secondary plasmalogen deficiency was reported in metabolic and inflammatory disorders such as diabetes mellitus, cardiac diseases, cancer, respiratory diseases and Alzheimer’s disease [3,4]. Secondary plasmalogen deficiency could result from decreased synthesis and/or increased degradation of plasmalogens. Replenishment and/or replacement of plasmalogens would be of substantial benefit in these diseases with plasmalogen deficiency.

Health effects of dietary phospholipids are recently reviewed [6], and the majority of studies indicated that dietary phospholipids have a positive impact in several diseases without severe side effect, however, dietary plasmalogen was not mentioned [6].

We prepared purified plasmalogens from chicken skin. We administered a diet containing 0.1% the purified plasmalogens to Zucker diabetic fatty (ZDF) rats and normal rats of Wistar strain. Routine laboratory tests of blood plasma (including total phospholipid) were done and phospholipid composition of plasma and erythrocytes were analyzed.

## Results and discussion

**Figure 1:**
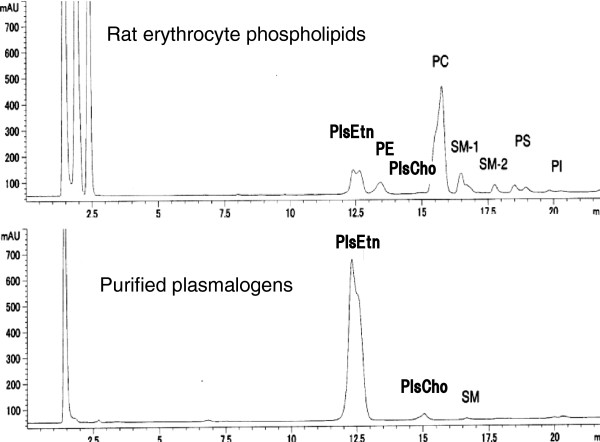
**HPLC chromatogram of the purified plasmalogen from chicken skin.** The retention times of ethanolamine plasmalogens (PlsEtn) and choline plasmalogen (PlsCho) are accorded to those of rat erythrocyte. The other abbreviations: PE, phosphatidylethanolamine; PC phosphatidylcholine; SM-1 and SM-2, sphingomyelin; PS, phosphatidyl serine; PI, phosphatidyl inositol.

The purified plasmalogens from chicken skin consisted of 96.4% ethanolamine plasmalogen (PlsEtn), 2.6% choline plasmalogen (PlsCho), 0.5% SM and 0.7% other phospholipids by calculation on the HPLC chromatographic area (Table 1, Figure 1). The retention times of chromatographic peaks of PlsEtn and PlsCho were accorded to those of PlsEtn and PlsCho of the rat erythrocyte membrane (Figure 1), and the peaks disappeared after treatment with hydrochloric acid (HCl), indicating the peaks were plasmalogens [7,8] (data are not shown). The fatty acids of the PlsEtn composed of DHA (22:6), ARA (20:4), oleic acid (18:1), myristic acid (14:0), palmitic acid (16:0) and stearic acid (18:0) (Table 1), which is in agree with that of ordinary plasmalogens found in mammalian tissues [1-4].

**Table 1:** Phospholipid composition of the purified plasmalogens and fatty acid composition of PlsEtn in the purified plasmalogens

**Phospholipid composition**		**Fatty acid composition of PlsEtn**	
		Fatty acid	
PlsEtn	96.4%	14:0	5.4%
PlsCho	2.6%	16:0	7.1%
SM	0. 5%	18:0	3.5%
LPE	0.7%	18.1	35.4%
		18:2	9.5%
		20:4	32.7%
		22:6	6.4%

Abbreviations; PlsEtn, ethanolamine plasmalogen; PlsCho, choline plasmalogen; SM, sphingomyelin: LPE, lysophosphatidylethanolamine.

**Figure 2:**
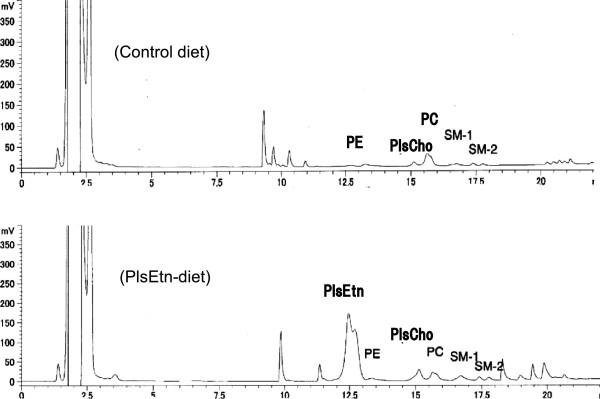
**HPLC chromatograms of phospholipids of the control diet and the plasmalogen diet (PlsEtn diet).** The chromatogram indicate that plasmalogens (PlsEtn and PlsCho) was actually contained in the PlsEtn diet. The other abbreviations: see Figure 1.

The chromatogram of phospholipid analysis of the PlsEtn diet showed a large peak of PlsEtn as compared to the control diet, indicating that PlsEtn was actually contained in the PlsEtn diet (Figure 2).

**Figure 3:**
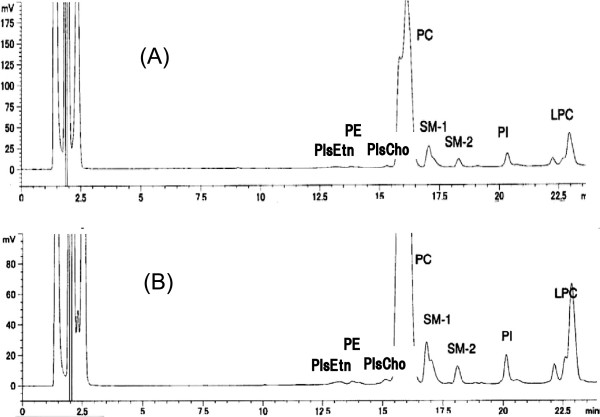
**A representative HPLC chromatogram of plasma phospholipids in Wistar rat.** Plasmalogens (PlsEtn and PlsCho) in rat plasma were detected by our method (**A**), but they were too small to evaluate the changes of these plasmalogens even though chromatograph was magnified (**B**). LPC, lysophosphatidylcholine; the other abbreviations, see Figure 1.

The diet contained 0.1 weight % the purified plasmalogens (PlsEtn diet) caused a decrease of plasma phospholipids as well as plasma cholesterol in ZDF rats within 4 weeks (Table 2). However, the other routine laboratory tests of plasma including triacylglycerol (TG), glucose, alanine aminotransferase (ALT), aspartate aminotransferase (AST), urea nitrogen and albumin were not different (Table 2). Body weight was also not different in the both diet groups. Phospholipid composition of the plasma analyzed by our HPLC method showed an increase of SM in the PlsEtn diet group (Table 3). Plasmalogens in the plasma was detected by our HPLC method, but they were too small to evaluate changes of plasmalogens (Table 3, Figure 3). The PlsEtn diet induced increases of erythrocyte PlsEtn and PE, and decrease of erythrocyte PC (Table 4).

**Table 2:** Changes in plasma cholesterol and phospholipids of ZDF rats after the PlsEtn for 4 weeks

	**Control diet (n = 10)**	**PlsEtn diet (n = 10)**	***t-test***
Total chol (mg/100mL)	206.3 ± 17.4	173.5 ± 15.3	*p < 0.001*
HDL-chol (mg/100mL)	60.3 ± 14.1	57.5 ± 9.6	
LDL-chol (mg/100mL)	9.2 ± 4.4	5.1 ± 2.4	*p < 0.02*
TG (mg/100mL)	1267.4 ± 367.9	1244.6 ± 361.2	
P-lipid (mg/100mL)	466.5 ± 35.5	409.3 ± 51.1	*p < 0.01*
Glucose (mg/100mL)	541.9 ± 172.8	507.5 ± 155.2	
Urea N (mg/100mL)	19.6 ± 1.6	19.0 ± 2.1	
AST (IU/L)	135.6 ± 94.2	126.5 ± 114.8	
ALT (IU/L)	96.1 ± 57.7	88.3 ± 83.9	
Albumin (g/100mL)	4.6 ± 0.3	4.5 ± 0.3	
Body weightt (g)	407.5 ± 30.9	391.5 ± 40.1	

Abbreviations: chol, cholesterol; HDL, high density lipoprotein; LDL, low density lipoprotein; TG, triacylglycerol; P-lipid, phospholipid; Urea N, urea nitrogen; AST, aspartate aminotransferase; ALT, alanine aminotransferase. Data are mean ± SD.

**Table 3:** Plasma phospholipid composition of ZDF rats after the PlsEtn diet for 4 weeks

	**Control diet (n = 10)**	**PlsEtn diet (n = 10)**	***t-test***
PlsEtn	0.39 ± 0.31	0.56 ± 0.26	
PE	0.46 ± 0.10	0.63 ± 0.22	
PlsCho	0.23 ± 0.11	0.35 ± 0.10	
PC	87.66 ± 1.14	86.88 ± 1.00	
PI	2.53 ± 0.25	2.55 ± 0.23	
SM	2.08 ± 0.47	2.67 ± 0.40	p < 0.01
LPC	5.60 ± 0.77	5.40 ± 0.84	

Data are % of total phospholipids calculated from chromatographic area detected by ELSD (mean ± SD).

**Table 4:** Changes in erythrocyte phospholipid composition of ZDF rats after the PlsEtn diet for 4 weeks

	**Control diet (n = 10)**	**PlsEtn diet (n = 10)**	***t-test***
PlsEtn	14.58 ± 0.76	15.65 ± 0.75	*p < 0.006*
PE	7.94 ± 0.54	8.82 ± 0.58	*p < 0.003*
PlsCho	0.39 ± 0.08	0.43 ± 0.07	
PC	62.69 ± 2.76	59.74 ± 1.53	*P < 0.001*
PS	4.21 ± 1.04	4.66 ± 0.47	
PI	0.91 ± 0.21	1.04 ± 0.15	
SM	9.27 ± 0.63	9.66 ± 0.52	

Data are % of total phospholipids calculated from chromatographic area detected by ELSD (mean ± SD).

The same PlsEtn diet was administered to normal rats of Wistar strain for 9 weeks. The PlsEtn diet again caused decrease of plasma phospholipid as well as plasma cholesterol, but the other routine laboratory tests of plasma were not changed (Table 5). Phospholipid composition of plasma was not different (Table 6). Phospholipid composition of the erythrocyte membranes showed an increase of PlsEtn and tendency to decrease of PC in the PlsEtn diet group (Table 7).

**Table 5:** Changes in plasma cholesterol and phospholipid of Wistar rats after the PlsEtn diet for 9 weeks

	**Control diet (n = 8)**	**PlsEtn- diet (n = 8)**	***t-test***
Total chol (mg/100mL)	98.4 ± 15.6	81.1 ± 10.2	*p < 0.05*
HDL-chol (mg/100mL)	53.6 ± 11.3	43.9 ± 7.8	*p < 0.07*
LDL-chol (mg/100mL)	22.1 ± 7.3	16.6 ± 5.5	*p < 0.10*
TG (mg/100mL)	145.1 ± 76.7	156.5 ± 73.7	
P-lipid (mg/100mL)	183.3 ± 22.9	152.7 ± 17.3	*p < 0.01*
Glucose (mg/100mL)	232.7 ± 36.8	220.9 ± 36.7	
Urea N (mg/100mL)	12.9 ± 1.9	13.1 ± 1.3	
AST (IU/L)	51.8 ± 4.3	53.4 ± 4.9	
ALT (IU/L)	24.9 ± 3.6	23.3 ± 3.7	
Albumin (g/100mL)	4.4 ± 0.2	4.3 ± 0.4	
Body weight (g)	478.8 ± 19.1	453.6 ± 21.5	

Abbreviations: chol, cholesterol; HDL, high density lipoprotein; LDL, low density lipoprotein; TG, triacylglycerol; P-lipid, phospholipid; Urea N, urea nitrogen; AST, aspartate aminotransferase; ALT, alanine aminotransferase. Data are mean ± SD.

**Table 6:** Plasma phospholipid composition of Wistar rats after the PlsEtn diet for 9 weeks

	**Control diet (n = 8)**	**PlsEtn diet (n = 8)**
PlsEtn	0.36 ± 0.18	0.41 ± 0.23
PE	0.34 ± 0.15	0.38 ± 0.17
PlsCho	0.43 ± 0.11	0.39 ± 0.05
PC	80.11 ± 1.24	79.95 ± 1.49
SM	5.78 ± 0.81	6.46 ± 1.65
PI	3.03 ± 0.29	2.63 ± 0.33
LPC	9.89 ± 0.84	9.73 ± 1.11

Data are % of total phospholipids calculated from chromatographic area detected by ELSD (mean ± SD).

**Table 7:** Change in erythrocyte phospholipids of Wistar rats after the PlsEtn diet for 9 weeks

	**Control diet (n = 8)**	**PlsEtn diet (n = 8)**	***t-test***
PlsEtn	15.76 ± 0.78	16.61 ± 0.83	*p < 0.03*
PE	8.45 ± 0.71	8.35 ± 0.49	
PlsCho	0.52 ± 0.12	0.51 ± 0.09	
PC	57.91 ± 2.04	56.71 ± 0.91	*p < 0.08*
PS	4.81 ± 0.92	4.91 ± 0.99	
PI	1.11 ± 0.21	1.21 ± 0.37	
SM	10.32 ± 1.09	10.64 ± 0.62	

Data are % of total phospholipids calculated from chromatographic area detected by ELSD (mean ± SD).

These results show that dietary PlsEtn induces decrease of plasma cholesterol and plasma phospholipids in normal rats as well as in ZDF rats. It has been known that dietary phospholipids cause decrease of plasma cholesterol probably due to inhibition of intestinal cholesterol absorption [6,9,10]. There is a report that dietary PE, but not dietary PC, caused a decreases of phospholipid, cholesterol and apolipoprotein A-1 in rat blood plasama [11]. Thus it is indicated that ethanolamine phospholipids, not limited to PlsEtn, induce a decrease of plasma phospholipid. Phospholipid composition of plasma in normal rats (Wistar rats) was not changed after 9 weeks with the PlsEtn diet (Table 6), and the other routine laboratory tests in plasma were not changed (Table 5). Body weight was also not different after 9 weeks of the PlsEtn diet (Table 5). These results indicate that dietary PlsEtn is not bad for health of normal rats despite the decrease of plasma phospholipids and cholesterol. It may be interesting to note that plasma triacylglycerol was not changed by the PlsEtn diet in both ZDF rats and Wister rats despite the decrease of cholesterol and phospholipid.

The PlsEtn diet to ZDF rats for 4 weeks induced increases of PlsEtn and PE, and decrease of PC in the erythrocyte membranes (Table 4). The PlsEtn diet to Wistar rats for 9 weeks also induced increase of PlsEtn and tendency to decrease of PC in the erythrocyte membrane (Table 7). Fatty acid composition of PlsEtn + PE showed an increase of ARA (20:4) and decrease of oleic acid (18:1) (Table 8). The increase of ARA in the erythrocyte membrane PlsEtn may reflect the fatty acid composition of PlsEtn in the diet (Table 1). These results of the erythrocyte membranes indicate that dietary PlsEtn can increase relative concentration of tissue PlsEtn.

**Table 8:** Fatty acid composition of erythrocyte phospholipids of Wistar rats after the PlsEtn diet for 9 weeks

**Fatty acid**	**Control diet**	**PlsEtn diet**	***t-test***
**[A] Fatty acid composition of erythrocyte PlsEtn + PE**			
22:6 + 14:0	5.46 ± 0.52	5.70 ± 0.38	
20:4	30.67 ± 4.89	35.06 ± 2.91	*p < 0.05*
18:2	10.21 ± 1.26	10.33 ± 0.50	
18:1	18.53 ± 2.29	17.69 ± 1.94	
16:0	23.17 ± 2.68	20.87 ± 1.60	*p < 0.06*
18:0	10.05 ± 2.13	8.02 ± 1.49	*p < 0.05*
other	1.92 ± 0.44	2.33 ± 0.81	
**[B] Fatty acid composition of erythrocyte PC**			
22:6 + 14:0	2.32 ± 0.41	2.41 ± 0.32	
20:4	8.63 ± 2.89	11.01 ± 1.71	*p < 0.07*
18:2	10.12 ± 1.12	11.76 ± 0.94	
18:1	11.63 ± 0.73	11.58 ± 0.47	
16:0	48.81 ± 3.44	45.64 ± 3.07	*p < 0.07*
18:0	16.21 ± 1.08	15.33 ± 1.30	
other	2.28 ± 0.41	2.29 ± 0.47	

Data are % of total fatty acids (mean ± SD).

Most of dietary glycerophospholipids may be hydrolyzed at the sn-2 position by pancreatic phospholipase A2 in the intestinal lumen and then absorbed by the enterocytes as free fatty acids and lysophospholipids [6,12]. The presence of plasmalogen-active phospholipase A2 in the small intestinal epithelium has been reported [13]. Because plasmalogens contain vinyl ether bond at the sn-1 position of glycerol, the lysophospholipid derived from dietary plasmalogens after digestion by phospholipase A2 is indicated to contain the vinyl ether bond. It is known that dietary alkyl glycerols are absorbed intact from the digestive tract without the cleavage of the ether bond [14-18]. However, whether the vinyl ether bond of dietary phospholipids absorbed intact from the intestine is not certain. The vinyl ether bond (1–0 alk-1’-enyl-sn-glycerol) is not same to ether bond of alkyl glycerols (1-0-alkyl-sn-glycerol). The vinyl ether bond is known to be especially sensitive to HCl [7,8]. There is a report, by using a diet containing 10% bovine brain phospholipids, that no degradation of plasmalogens under in vitro conditions simulating those of the stomach and small intestinal lumen was observed [19], and they also reported that ingestion of 10% bovine brain phospholipids increased plasmalogen in blood plasma [19]. Thus it is likely that lysophospholipids (lysoplasmalogens) containing the vinyl ether bond derived from the dietary PlsEtn are absorbed intact from rat intestine.

Since 1-0-alkylglycerol enter the plasmalogen biosynthesis pathway downstream of the peroxisomal step, plasmalogen replacement therapy for inherited peroxisomal disorders by using dietary 1–0 alkylglycerols are reported and the therapy induced increases of plasmalogens in several tissues [15-18,20]. Wood et al. [20,21] synthesized an alkyl-diacyl plasmalogen precursor with palmitic acid at sn-1, DHA at sn-2 and lipoic acid at sn-3 (PPI-1011). They reported that PPI-1011 replenished PlsEtn of cultured lymphocytes from RCDP patients [20], and the oral administration of PPI-1011 to rabbits for 2 weeks increased DHA and DHA-containing PlsEtn in plasma and retina [21].

The PlsEtn diet to ZDF rats increased relative composition of PlsEtn of erythrocyte membranes within 4 weeks, and the same diet to normal rats for 9 weeks also increased relative composition of PlsEtn of the erythrocytes. The present study indicates that dietary PlsEtn is safe for health of normal rats despite the decrease of plasma phospholipids. Oral administration of purified plasmalogens may be more efficient for replenishment and/or replacement of plasmalogens of tissues than that of alkyl glycerols or alkyl-diacyl plasmalogen precursor (PPI-1011), because administration of plasmalogen may supply glycerol with vinyl ether bond, ethanolamine, choline and polyunsaturated fatty acids such as DHA and ARA, all of these substances may be re-used to biosynthesis of plasmalogens at various tissues.

Recently, we reported that pre-treatment with the purified plasmalogen from chicken breast muscle (composed 47.6% PlsEtn and 49.3% PlsCho) by intraperitoneal administration attenuate lipopolysaccharide-induced neuroinflammation [22]. However, whether dietary plasmalogens increase plasmalogen content of brain with plasmalogen deficiency remain to be tested.

## Materials and methods

### Preparation of purified plasmalogens

Plasmalogens were prepared from chicken skin. The chicken skin was obtained from a market place. Most of fat of the chicken skin was initially removed by a steam oven, then, the skin was freeze dried. Plasmalogens were purified from the freeze dried skin essentially by the procedure as reported previously [23].

### Animals and diets

Diet containing 0.1 weight % purified plasmalogens (PlsEtn diet) was prepared by supplementation of the purified plasmalogens to control diet (AIN 96). These diets were prepared and supplied by Oriental Yeast Co (Tokyo, Japan).

All of the experiments involving the use of animals were accorded to the Guiding Principles for the Care and Use of Animals of the Physiological Society of Japan, and all efforts were made to minimize animal suffering and the number of animals used for the experiments.

All rats were obtained from KBT Oriental Co. (Tosu City, Saga Prefecture, Japan). Twenty male rats of ZDF rats, aged 4 weeks, received the control diet for 10 days, and they were divided into two groups at random. One group (10 rats) was fed the control diet for an additional 4 weeks (control diet group), and the other groups (10 rats) was fed the PlsEtn diet (PlsEtn diet group) for 4 weeks. In another experiment, 18 male rats of Wistar strain, aged 6 weeks, received the control diet for 10 days, and divided into two groups. One group received the control diet for additional 9 weeks (control diet group) and the other group received the PlsEtn diet for 9 weeks. All rats were housed in stainless steel wire cages in a air conditioned room (22°C) with 12 hour cycle of light and dark. They had free access to water and the diets.

### Preparation of erythrocytes

The rats were deprived of food for 14 to 16 hours, and, after ether anesthesia, blood was collected into tube containing EDTA by cardiac puncture. Blood plasma was separated by centrifugation at 1000 x g for 5 min. Erythrocytes were prepared after the collection of plasma. After removing the buffy coats and small portion of top layer of erythrocytes, the erythrocytes were washed three times in cold isotonic saline at 1000 g for 5 min at 4°C. A small portion of the top layer was removed at each washing.

### Biochemical analysis of plasma

A part of the plasma was sent to a clinical laboratory center (CRC Co, Fukuoka, Japan) and routine clinical laboratory tests of plasma including plasma phosholipid were done using an automated analyzer (Olympus AU-5200), in which total cholesterol, total phospholipid and triacylglycerol were determined by each enzymatic method (Cholsterol-C test, Phospholipid-B test and Triglyceride-G test, Wako Pure Chemical Co Osaka, Japan).

### Extraction of total lipids

Extraction of total lipids from erythrocytes was done immediately after the preparation of the washed erythrocytes [8,23,24]. Briefly 500 μL of the packed erythrocytes was hemolyzed with equal volume of water. Four mL of methanol was added to lysate followed after 40 min by 4 mL chloroform. After an additional 30 min, the extract was centrifuged and residue was re-extracted with 4 mL of chloroform/methanol (1:1). Pooled extracts were washed with 10 mL 0.88% KCl to make biphasic mixture. Two mL of lower phase was dried under N2 gas. Extraction of total lipid from plasma (500 μL) was also accorded to that from the erythrocytes. Methanol and chloroform used in the extraction of lipids contained butylhydroxytoluene (50 mg/L).

### Separation of phospholipid classes

Separation of phospholipid classes including plasmalogens (Pls) was done by our reported method [8]. The HPLC system used was an Agilent HPLC system (HP-1100 Series, Agilent Technologies, Tokyo, Japan) equipped with an evaporative light scattering detector (ELSD), a fluorescence detector and a UV dector. The system was connected to a Chem Station (Agilent Technology) for control and analysis of chromatograms. Relative composition of phospholipid classes were measured on each chromatographic area with ELSD detection.

### Analysis of fatty acid composition of phospholipids

Each phospholipid was collected from an HPLC with UV detection [23,24], and analysis of fatty acid composition of phospholipids was accorded to our previous reports [23,24].

### Data analysis

Data were analyzed by using a paired *t* test (two tailed) with p < 0.05 used for significance.

## Competing interests

The authors declare that they have no competing interests.

## Authors’ contributions

SM operated all experiments and wrote the manuscript.TK participated in critical discussion. KM prepared the purified plasmalogens. TF designed the study and participated in critical discussion. All authors read and approved the final manuscript.
